# A Retrospect of Christmas at Bristol

**Published:** 1913-01-11

**Authors:** 


### CHRISTMAS IN THE INSTITUTIONS.

### A RETROSPECT OF CHRISTMAS AT BRISTOL.

We all had a very good time this Christmas at the Bristol General Hospital. And we are feeling rather pleased with ourselves. Even a selfish person can get a considerable amount of solid satisfaction out of trying to do a little good for others. Even the laziest of us had a shot this year, and the result is apparently not unsatisfactory. Now for details. It is an old-fashioned hospital in some ways, the "General" (what would our up-to-date committee say to this?), and we cling rather to customs and traditions instituted in the ignorant "pre-us" period. This point is borne out in the ritual of the seven days or so surrounding Christmas. No attempt was made to strike out on fresh lines. The old ones had done very well. The decorations were ornate and tasteful, as the lay newspapers say. One ward had in a very clever way been converted into a veritable bower of roses. "Roses all the way," they even climbed up the beds. The effect was charming. "Casualty" was a dream of ivy and electric lighting. Cunning little lamps in the shape of peaches, strawberries, etc., peeped out everywhere, and the ex-ophthalmic appearance of the out-patients as they entered was good to behold. The "picture-palace" motto, "This Show never Stops," flaunted itself in scarlet and gold over an arch facing the entrance.

We began to be "Christmassy" on Christmas Eve. The old custom of singing carols in the courtyard was reluctantly abandoned. As it simply poured with rain, this was perhaps wise. Christmas Day was revelry from the word "Go." The sight of the heavy-weight senior house surgeon carving turkeys and geese in the wards was one to linger in the memory. Mind you, he got those birds dissected in time. Something had to go. What is a bird compared with a man! He is leaving us now to serve his country in India, and he will always be remembered as one of the best. The day was a phantasmagoria of food, carols, and smiles. Everybody was glad to get to bed, but they did not admit it till they got there. Boxing Day—a concert for the patients. The nurses' lounge and lecture-room were thrown into one large hall by the withdrawal of screens, a beautifully decorated stage prepared by the excellent hospital carpenter, with some outside assistance, and the place packed with patients and nurses. Quite a short programme. First half, miscellaneous—songs of various kinds, instrumental duets, and some delightful recitations by Nurse Irene Keys. "Better than you'd hear on the stage," say the patients, and we thought so too. Then a farce, called "Chiselling." Oh, those rehearsals! How we did try to be like "real actors." Well, it went down all right. A senior surgical nurse was a portly landlady; a rather juvenile-looking house surgeon was a fussy old gentleman. And well they did it! Everyone says Mr. Bolt has mistaken his vocation. The audience agreed, too, that the heroine and the slavey looked "simply sweet." Next night we did it again, but this time the lights shone on fair women and brave men. Half Clifton was there looking its bonniest. "Do you think it will go off all right?" we asked each other in whispers in the "green room" (Oh, yes, we had a green room!) Fortunately all was well, and the Matron informed us that we were a success. We breathed again and reflected how much of the credit was due to the untiring work of the Home Sister behind the scenes.

Saturday—there was "Casualty" Christmas tree. Hundreds of the poorest children of the neighbourhood, who had qualified by minor injuries for an appearance, assembled in the courtyard. On admission what a sight met their eyes! A huge Christmas tree blazing with electric light and crammed with toys, the handsome "Casualty" house surgeon disguised as Father Christmas (how splendid he looked!), Sister "Casualty" with her most winning smile, and a small army of helpers. Then followed an ecstatic five minutes. By devious ways the juvenile visitors were conducted round and received in turn a toy, an orange, a bag of sweets, and a cup of cocoa, and then out into the cold world again. You never saw such a crowd. Oh! the tragic look on Sister's face when the supply ran short and there were still some children left! The Matron, like a good fairy, produced more gifts, and all were sent home happy. In the evening the servants had their party.

Monday, December 30, another Christmas tree. This time a different sort of affair. The gentle exercise known as "pulling the leg" was much indulged in. In the words of the reporters:—"The gifts were of a personal and appropriate character" and the individual with an idiosyncrasy found some special tribute awaiting him on the tree. Then in the evening the Christmas season wound itself up by a Coon Concert. The residents and students, in fearful and wonderful attire, went round the wards and sang to the patients. Poor patients, they couldn't escape. However, they stood it all right and politely said they liked it. And so to work again. It was a good time, and we are almost sorry it's over.

